# The Severity of the Co-infection of Mycoplasma pneumoniae in COVID-19 Patients

**DOI:** 10.7759/cureus.24563

**Published:** 2022-04-28

**Authors:** Rohit Rangroo, Myles Young, Alexander Davis, Steven Pack, Shaival Thakore, Anna Schepcoff, Olu Oyesanmi

**Affiliations:** 1 Internal Medicine, HCA Florida Bayonet Point Hospital, Hudson, USA; 2 Internal Medicine, Oak Hill, Tampa, USA

**Keywords:** covid-19 co-infection, pulmonary critical care, mycoplasma pneumonia, acute respiratory distress syndrome [ards], sars-cov-2 (severe acute respiratory syndrome coronavirus -2), covid-19

## Abstract

Background and objective

The severe acute respiratory syndrome coronavirus 2 (SARS-CoV-2) is a novel coronavirus that causes coronavirus disease 2019 (COVID-19) infection, with symptoms ranging from mild upper respiratory illness to multisystem organ failure, and even death. Since its discovery in December 2019, the SARS-CoV-2 virus has led to a global pandemic, rapidly spreading to countries around the world, with millions of reported deaths to date. As researchers around the world continue to analyze and interpret the data gathered regarding the novel virus, it is evident that its co-infection with various bacterial pathogens is associated with a worse overall prognosis. One such bacterial pathogen, *Mycoplasma pneumoniae* (*M. pneumoniae*), has been associated with an increase in inpatient mortality, length of hospital stay, and need for mechanical ventilation. The aim of this study was to evaluate the characteristics and outcomes of patients co-infected with SARS-CoV-2 and *M. pneumoniae*. We sought to determine if this co-infection led to increased incidence of ventilatory support, intensive care unit (ICU) stay, and mortality.

Materials and Methods

A multi-center retrospective study was conducted involving patients aged 18 years and older. We compared the incidence of in-hospital mortality, ICU stay, and mechanical ventilation support between COVID-19-positive patients with and without *M. pneumoniae* co-infection. Based on the collected data, a binary logistic regression model was implemented to assess the correlation between mortality and ventilatory support, while linear regression was used to study the length of stay (LOS) independent variable.

Results

A total of 1,208 patients with a positive SARS-CoV-2 test were identified. Among them, 604 (50%) had an *M. pneumoniae* co-infection. LOS (95% CI for the coefficient estimate [0.86, 1.05], p<0.001), need for mechanical ventilation (95% CI for the odds ratio [2.60, 6.02], p<0.001), and inpatient mortality (95% CI for the odds ratio [1.43, 2.97], p<0.001) among those co-infected were significantly higher compared to COVID-19 patients without concomitant *M. pneumoniae* infection.

Conclusion

COVID-19 with a concomitant *M. pneumoniae* infection was found to have worse outcomes and overall prognosis when compared to individuals with independent disease states. Based on retrospective data gathered from a large multicenter database, the rates of mortality, ventilatory support, and length of hospital stay were significantly worse in patients with a co-infection of SARS-CoV-2 and *M. pneumoniae*.

## Introduction

The severe acute respiratory syndrome coronavirus 2 (SARS-CoV-2) is a novel coronavirus and the causative agent of coronavirus disease 2019 (COVID-19). Since its emergence in Wuhan, China in December of 2019, the virus has spread rapidly across the world, resulting in a global pandemic. COVID-19 patients who require mechanical ventilation are often determined to have an overall poor prognosis. Often complicated by additional pulmonary manifestations, such as acute respiratory distress syndrome (ARDS), pneumothorax, pneumomediastinum, and pulmonary embolism, COVID-19 leads to significant ventilatory dependence. Unfortunately, pulmonary diseases are not the only manifestations of COVID-19 infection. As mentioned in a study by Finsterer et al., the most frequent extrapulmonary manifestations of COVID-19 include hyposmia, hypogeusia, abdominal pain, nausea, diarrhea, vomiting, hepatopathy, and thrombosis [1].

On March 11, 2020, the World Health Organization (WHO) declared COVID-19 a global pandemic. Since then, over 246 million cases worldwide and more than five million deaths have been reported by the Centers for Disease Control and Prevention (CDC). As of January 2022, over 45 million cases and 740,000 deaths have been reported in the United States alone, and these rates continue to be on the rise [2,3]. As reported by previous studies, it is estimated that 50% of patients who had died of COVID-19 had various bacterial co-infections, complicating their hospital course and prognosis [4,5]. Additionally, it is understood that these co-infections increase the overall clinical severity by increasing morbidity, mortality, length of stay (LOS) in hospitals, intensive care unit (ICU) admission, and the need for aggressive respiratory support including mechanical ventilation [6]. Specifically, a recent study has outlined the most common co-infections identified with SARS-CoV-2, including *Streptococcus pneumoniae*, *Staphylococcus aureus*, *Klebsiella pneumoniae*, *Haemophilus influenzae*, *Mycoplasma pneumoniae (M. pneumoniae),* *Acinetobacter baumannii*, *Legionella pneumophilia*, and *Chlamydia pneumoniae* [7]. Of these co-infections, *M. pneumoniae* has had the strongest association with SARS-CoV-2 [8]. A meta-analysis has found that 7% of hospitalized COVID-19 patients had coexisting bacterial infections, 14% of which were in the ICU setting, with *M. pneumoniae* being the leading bacterial pathogen [8]. This accounted for 42% of all co-infection cases reviewed [8]. *M. pneumoniae* has been identified as the most commonly associated co-infection, and it is important to further identify the changes in outcomes and risks associated with it. Much like the 1918 H1N1 Spanish Flu pandemic, the morbidity and mortality among COVID-19 patients with bacterial co-infection are significantly higher [9]. These higher outcomes were once again observed during the 2009 H1N1 Swine Flu pandemic with documented co-infection rates as high as 23% [9,10]. This study seeks to examine the severity of SARS-CoV-2 with *M. pneumoniae* co-infection to better understand the complications of COVID-19 with comorbidities and its impact on patient outcomes.

SARS-CoV-2 is a betacoronavirus belonging to the subfamily of coronaviruses that have been responsible for larger outbreaks including SARS and the Middle East respiratory syndrome (MERS) [11]. When compared to the coronaviruses causing SARS or MERS, SARS-CoV-2 is much more virulent, which is likely related to the host's ability to produce T-cells. Factors such as the viral load, route of infection, inoculation density, age, and immune status of the patients also contribute to its severity and complications [11]. Particularly, its known destruction of type II pneumocytes and subsequent macrophage recruitment lead to large increases in interleukin-1 (IL-1), interleukin-6 (IL-6), and tumor necrosis factor-ɑ (TNF-α) [11]. Due to this robust inflammatory response, resulting in severe disease and poor outcomes, the severity associated with SARS-CoV-2 has ranged from asymptomatic infection to death. Although most patients have only mild symptoms requiring supportive care, one ICU case series consisting of 21 patients has reported 17 of them requiring invasive mechanical ventilation, due to the development of severe ARDS, with six patients ultimately expiring [12].

*M. pneumoniae* is a small bacterial pathogen lacking a peptidoglycan wall, which was first discovered in the 1940s, often resulting in atypical pneumonia. Of the various pathogens associated with community-acquired pneumonia, *M. pneumoniae* is a leading cause, detected in approximately 5.2-27.4% of all cases [13]. Apart from being a leading cause of pneumonia, *M. pneumoniae* has also been associated with pathologies seen in multiple organ systems, including cardiovascular, gastrointestinal, neurological, integumentary, hematological, and renal [14]. Extrapulmonary manifestations occur through local inflammation via cytokines and distant inflammation via immune modulation [14]. The complex pathogenesis of *M. pneumoniae* is often due to the direct and immune-related damage. Direct damage occurs through the respiratory epithelium as well as fibroblasts and macrophages in vitro. This process of adhesion relies heavily on P1 protein attachment, resulting in a cascade of invasive and membrane fusion damage, nutrition depletion, and toxic necrosis. The toxic necrosis can lead to cellular swelling, cell damage, slowing of microvilli movement, and structural deformation, thereby precipitating an increase in lymphocyte, plasma cell, and monocyte activity as well as the production of oxidative stress [15,16]. The immune damage includes humoral, antigen immune, inflammatory, and immunosuppression activation, with a known increase in the activity of TNF-ɑ, IL-1B, and IL-6 [17].

## Materials and methods

A multi-center retrospective study was conducted based on an HCA enterprise-wide database within the United States, covering the period from January 1, 2020, to March 1, 2020. The inclusion criteria were as follows: patients aged 18 years and above who were diagnosed with COVID-19 by reverse transcription-polymerase chain reaction (RT-PCR) or rapid antigen testing with and without a co-infection diagnosis of *M. pneumoniae* by positive IgM titers. Demographic data including age, sex, race, body mass index (BMI), diagnosis of diabetes mellitus, diagnosis of chronic obstructive pulmonary disease (COPD), and history of tobacco use were gathered (Table 1).

**Table 1:** Summary of the demographic data CI: confidence interval; BMI: body mass index; COPD: chronic obstructive pulmonary disease

Characteristics	Values
Age, years	
Min	18
Mean (95% CI)	59.73 (58.7, 60.76)
Median	61
Max	90
Sex, n (%)	
Male	629 (52.1%)
Female	579 (47.9%)
Race, n (%)	
White	688 (57.0%)
Black	254 (21.0%)
Asian	27 (2.2%)
Other/unknown	239 (19.8%)
BMI, kg/m^2^	
Min	12.77
Mean	30.20
Median	29.25
Max	49.88
Diabetes, n (%)	
Yes	415 (34.4%)
No	793 (65.6%)
Tobacco use, n (%)	
Yes	4 (0.3%)
No	1,204 (99.7%)
COPD, n (%)	
Yes	12 (1%)
No	1,196 (99%)

## Results

A total of 1,208 patients (*M. pneumoniae* IgM-positive: 50%, *M. pneumoniae* IgM-negative: 50%), with a minimum age of 18 and maximum age of 90 years (mean age of 59.73 years [95% CI: 58.7, 60.8]), were included.

The odds of inpatient mortality among patients with *M. pneumoniae* co-infection were approximately twice as high as those who did not have *M. pneumoniae* co-infection (95% CI for the odds ratio [1.43, 2.97], p<0.001) (Table 2). For every one-year increase in patient age, there was a corresponding 6% increase in the odds of inpatient mortality (95% CI for the odds ratio [1.04, 1.07], p<0.001). Interestingly, the odds of inpatient mortality among female patients were 40% lower than those of male patients (95% CI for the odds ratio [0.42, 0.86], p=0.01).

**Table 2:** Mortality rates in COVID-19 patients with various comorbidities COVID-19: coronavirus disease 2019; CI: confidence interval; BMI: body mass index; COPD: chronic obstructive pulmonary disease

Binary logistic regression for mortality			
Model term	Coefficient estimate (95% CI)	Odds ratio (95% CI)	P-value
(Intercept)	-6.81 (-8.38, -5.31)	0.00 (0.00, 0.00)	<0.001
Age	0.06 (0.04, 0.07)	1.06 (1.04, 1.07)	<0.001
Sex = female	-0.51 (-0.87, -0.15)	0.60 (0.42, 0.86)	0.01
Race = black	-0.12 (-0.63, 0.36)	0.89 (0.54, 1.43)	0.63
Race = Asian	-0.35 (-2.22, 0.97)	0.71 (0.11, 2.63)	0.65
Race = other/unknown	0.42 (-0.03, 0.86)	1.52 (0.97, 2.36)	0.06
BMI	0.03 (0.00, 0.06)	1.03 (1.00, 1.06)	0.07
Diabetes	0.58 (0.22, 0.93)	1.78 (1.25, 2.54)	<0.001
Tobacco use	-12.22 (0.00, 37.03)	0.00 (0.00, 1.20E+16)	0.98
COPD	-0.95 (-3.89, 0.77)	0.39 (0.02, 2.16)	0.37
Mycoplasma pneumoniae	0.72 (0.36, 1.09)	2.05 (1.43, 2.97)	<0.001

With regard to LOS, patients with *M. pneumoniae* co-infection had LOS that was 159% higher (i.e., approximately 2.5 times higher) than those without *M. pneumoniae* co-infection (95% CI for the coefficient estimate [0.86, 1.05], p<0.001) (Table 3). For every 10-year increase in patient age, on average, we observed an approximate 20% increase in patient LOS (95% CI for the coefficient estimate [0.015, 0.021], p<0.001).

**Table 3:** Length of stay for COVID-19 patients with various comorbidities COVID-19: coronavirus disease 2019; LOS: length of stay; CI: confidence interval; BMI: body mass index; COPD: chronic obstructive pulmonary disease

Linear regression for log LOS		
Model term	Coefficient estimate (95% CI)	P-value
(Intercept)	-0.35 (-0.67, -0.02)	0.04
Age	0.02 (0.015, 0.021)	<0.001
Sex = female	-0.19 (-0.28, -0.09)	<0.001
Race = black	-0.01 (-0.14, 0.11)	0.85
Race = Asian	0.24 (-0.09, 0.57)	0.16
Race = other/unknown	0.03 (-0.09, 0.16)	0.61
BMI	0.01 (0.01, 0.02)	<0.001
Diabetes	0.32 (0.21, 0.42)	<0.001
Tobacco use	0.34 (-0.50, 1.17)	0.43
COPD	-0.03 (-0.51, 0.46)	0.92
Mycoplasma pneumoniae	0.95 (0.86, 1.05)	<0.001

Patients with *M. pneumoniae* co-infection had odds of mechanical ventilation that were approximately 3.91 times as high as patients who did not have *M. pneumoniae* co-infection (95% CI for the odds ratio [2.60, 6.02], p<0.001) (Table 4). Also, for every one-year increase in age, there was a corresponding 3% increase in the odds of the patient being placed on a ventilator (95% CI for the odds ratio [1.01, 1.04], p<0.001).

**Table 4:** Rates of mechanical ventilation in COVID-19 patients COVID-19: coronavirus disease 2019; CI: confidence interval; BMI: body mass index; COPD: chronic obstructive pulmonary disease

Binary logistic regression for mechanical ventilation			
Model term	Coefficient estimate (95% CI)	Odds ratio (95% CI)	P-value
(Intercept)	-6.75 (-8.28, -5.29)	0.00 (0.00, 0.01)	<0.001
Age	0.03 (0.01, 0.04)	1.03 (1.01, 1.04)	<0.001
Sex = female	-0.74 (-1.13, -0.37)	0.47 (0.32, 0.69)	<0.001
Race = black	0.14 (-0.34, 0.60)	1.15 (0.71, 1.82)	0.56
Race = Asian	0.96 (-0.54, 2.14)	2.62 (0.58, 8.51)	0.15
Race = other/unknown	0.13 (-0.35, 0.60)	1.14 (0.70, 1.83)	0.58
BMI	0.07 (0.04, 0.10)	1.08 (1.05, 1.11)	<0.001
Diabetes	0.49 (0.12, 0.86)	1.64 (1.13, 2.37)	0.01
Tobacco use	-14.34 (-14.34, 118.48)	0.00 (0.00, 2.87E+51)	0.99
COPD	-14.31 (-14.31, 32.46)	0.00 (0.00, 1.25E+14)	0.98
Mycoplasma pneumoniae	1.36 (0.96, 1.80)	3.91 (2.60, 6.02)	<0.001

## Discussion

We believe that during the height of the COVID-19 pandemic, investigations into co-infections may have been lower than expected due to the utmost sense of urgency among the medical community to identify and treat COVID-19 to decrease its morbidity and high mortality rates. In this retrospective study, we have found that a co-infection with *M. pneumoniae* significantly correlates to increased patient overall mortality, LOS, and need for mechanical ventilation among COVID-19 patients.

Patient mortality was approximately two times higher in COVID-19 patients having co-infection with *M. pneumoniae*. Based on the co-infection rates of other large-scale infections of the past, we suspect that it would also apply to the ongoing COVID-19 scenario as well [9,10]. Additionally, an increase in age was associated with increased mortality among our cohort. We found that every one-year increase in patient age was associated with a corresponding increase in the odds of inpatient mortality. As we age, our immune response declines to a low-grade pro-inflammatory state, accompanied by suboptimal innate immune responses [18].

Our study also identified that gender played a role in the severity of illness, as mortality was found to be 40% lower in women than in men. Specifically, regarding gender, there is data indicating that estrogen receptor function and activation occur across many immune cells including T cells, B cells, natural killer cells, macrophages, dendritic cells, and neutrophils, as well as cytokine modulation and B cells activation [18]. This contrasts with androgens and progesterone, which act as immune suppressors. These are known to increase IL-4 and lower interferon-gamma (IFN-γ) helper cell type 1 responses [18].

When looking into the potential synergy of *M. pneumoniae* and SARS-CoV-2, it was found that both target the lungs at similar locations, and cause large inflammatory responses [11,17]. They both attack the lung respiratory epithelial layer similarly. We believe that this provides an opportunity for co-infection between pathogens and increases the severity of illness. This severity of COVID-19 illness has led to intubation and longer LOS as seen in our data, showing that mechanical ventilation occurred 3.91 times higher in cases of co-infection. Typically, whenever invasive airway protection is needed, we would expect to see an increase in LOS. What we, unfortunately, cannot determine is the timing of *M. pneumoniae* infection in relation to infection with SARS-CoV-2. It may be of future interest to see if one precipitates the other. Interestingly, another study has led to the inclusion of azithromycin, an antibiotic that is frequently used in the treatment of *M. pneumoniae* infections, as the standard-of-care treatment for COVID-19 infection [11]. This research has likely benefited patients with SARS-CoV-2 who had not been tested for *M. pneumoniae* co-infection.

One of the major limitations of this retrospective study was the under-representation of tobacco users and those with concomitant diagnoses of COPD. Both of these characteristics were present in less than 1% of the population and were not sufficiently analyzed in the study. It is unknown whether or not tobacco use and COPD have a significant impact on the severity of COVID-19. It is also important to note that the average BMI in this study was high, with a mean BMI of 30. Previous studies have already identified an association between a higher BMI and COVID-19 severity [18]. Also, co-infection with other virulent pathogens, such as *Streptococcus pneumonia*, *Staphylococcus aureus*, *Klebsiella pneumoniae*, *Haemophilus influenzae*, *Acinetobacter baumannii*, *Legionella pneumophilia*, and *Chlamydia pneumoniae,* were not analyzed. Just as co-infection with *M. pneumoniae* is significantly associated with worsening of SARS-CoV-2 infection, it is possible that these other pathogens may be underdiagnosed and causing similar outcomes.

Additional limitation pertains to the provider ordering practices for *M. pneumoniae* testing and treatment heterogenicity. Prior to the COVID-19 pandemic, the ordering of markers for atypical pneumonia was based on clinical suspicion regarding the need to identify a causative organism. Throughout this pandemic, it has been difficult to determine the utility of ordering additional testing after receiving positive SARS-CoV-2 results. This concern could have affected the results found in our study sample, indicating that co-infection cases could be missing from our data. Another viewpoint is that in worsening cases, there was perhaps a desire to test for other compounding infections, which may skew more severe cases as those with co-infections, with others not being accounted for.

As more data continue to be available regarding the SARS-CoV-2 virus, it is evident that co-infection with various bacterial and viral pathogens is quite prevalent and often impacts the overall prognosis [19].

## Conclusions

Clinicians should be mindful of and evaluate for co-infections, particularly *M. pneumoniae*, in COVID-19 patients. Further research can expand on our findings and seek ways to improve SARS-CoV-2 morbidity and mortality. It is evident that *M. pneumoniae *co-infection in COVID-19 patients leads to higher mortality rates, longer hospitalizations, and increased rates of mechanical ventilation.
